# Sex and Age Differences in Habitat Selection of the Mountain Dragon Lizard (*Diploderma splendidum*) From Western China

**DOI:** 10.1002/ece3.70724

**Published:** 2024-12-23

**Authors:** Dongqing Zheng, Ling Li, Wei Gao, Meiqi Chen, Peng Guo, Yayong Wu

**Affiliations:** ^1^ Faculty of Agriculture, Forest and Food Engineering Yibin University Yibin Sichuan China; ^2^ School of Life Science and Technology Xinjiang University Urumqi Xinjiang China; ^3^ State Key Laboratory of Genetic Resource and Evolution & Yunnan Key Laboratory of Biodiversity and Ecological Security of Gaoligong Mountain, Kunming Institute of Zoology Chinese Academy of Sciences Kunming Yunnan China

**Keywords:** age differences, *Diploderma splendidum*, habitat selection, sex differences, Western China

## Abstract

Habitat selection in animals results from a careful balance of individual requirements, environmental conditions, and ecological disturbances. Preferences can vary across sexes and ages due to differences in survival and reproductive priorities. Despite this variability, most studies have traditionally focused on isolated aspects of either sex or age‐related differences in habitat selection, rather than considering a comprehensive range of influencing factors. The mountain dragon lizard (*Diploderma splendidum*) exemplifies a species adapted to shrub habitats in the dry‐hot river valleys of the lower Jinsha River, Western China, playing a crucial role in regional ecosystem stability. In this study, we examined the influence of 11 ecological factors on habitat selection by male and female *D. splendidum* across two distinct age classes (adult and juvenile) to explore sex and age‐related disparities. The lizards showed considerable similarity in habitat preferences, but notable differences in their selection of specific ecological factors. Compared to adult females, adult males displayed a preference for higher tree positions, lower light intensity, and moderate vegetation density. Compared to juvenile females, juvenile males favored higher tree positions, low rock formations, and shrubby grassland and forest. Compared to juvenile females, adult females preferred higher tree positions and habitats further from water. Compared to juvenile males, adult males preferred higher tree habitats. Overall, habitat selection complexity in *D. splendidum* was significantly influenced by sex and age factors. This study contributes to our understanding of how these lizards respond to different physiological structures and resource requirements. These findings enhance current knowledge on reptile habitat selection and provide theoretical insights crucial for ecological restoration and species protection in the hot and dry valley areas of Hengduan Mountain.

## Graphical abstract

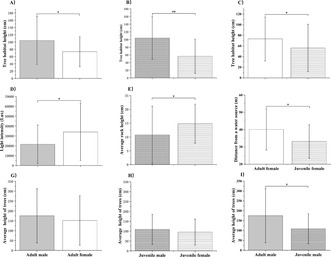

## 1 Introduction

Selection of suitable habitats is a critical process for animal survival and reproduction (Danchin, Boulinier, and Massot 1998). Habitat quality significantly influences resource utilization among species, as resources are often unevenly distributed, compelling all species to compete for the most suitable habitats to maximize resource acquisition (Paterson and Blouin‐Demers 2018). Consequently, the factors shaping wildlife habitat selection are complex, encompassing both biotic and abiotic elements (Reunanen, Mönkkönen, and Nikula 2002). These factors may arise from distinct physiological structures or phenotypic adaptations, including the intricate interplay between structure and function (Kaliontzopoulou, Carretero, and Llorente 2010; Popova et al. 2021). They also encompass dynamics of inter‐ and intraspecific competition, antipredator behavior, thermoregulatory responses in different environments and external forces such as seasonal changes or initiation of breeding seasons (Seki and Sato 2022; Li et al. 2022; Bergstrom et al. 2019; Gaudenti et al. 2021; Ivey et al. 2020). Thus, habitat characteristics affecting fitness may vary spatially or temporally depending upon the phenotype of individuals or prevailing environmental conditions (Delaney and Warner 2016).

Sexual dimorphism often leads to distinct habitat preferences among individuals due to physiological disparities and variations in life history strategies (Boinski 1988; Calsbeek 2009). Males typically exhibit greater habitat flexibility, investing more in selecting habitats suitable for social displays or territorial defense (Gabbert et al. 1999; Delaney and Warner 2016). Conversely, females typically prioritize habitats with reduced security threats and enhanced predation avoidance strategies, with a preference for habitats conducive to oviposition, parturition escape, and concealment (Hahn and Silverman 2006; Kidawa and Kowalczyk 2011). Recknagel et al. (2023) discovered that female common lizard (*Zootoca vivipara*) exhibited a preference for habitats characterized by higher tree density and lower ground exposure compared to males, likely due to their reduced mobility during pregnancy. Optimal habitat for pregnant and lactating females is characterized by lower predator presence and abundant food resources, crucial for promoting reproductive success and offspring fitness (Bongi et al. 2008; Rachlow and Bowyer 1998). Zhao and Liu (2012) found that female agamid lizards ( *Phrynocephalus przewalskii* ) favor habitats with lower vegetation cover to regulate body temperature, but after breeding, they prefer habitats with high grass density and cover to reduce threats and accumulate energy. Overall, differences in biological functions, social responsibilities, and division of labor between the sexes have resulted in varying degrees of habitat selection preferences among individuals of both sexes (Delaney and Warner 2016; Zhao and Liu 2012).

Differential habitat selection can also result from varying resource needs across different developmental stages, leading to distinct patterns of selection (Barten, Bowyer, and Jenkins 2001). Adults typically prioritize habitats that facilitate sexual communication and territorial defense, essential for breeding (Boinski 1988). In contrast, juveniles often seek out locations with reduced competition and increased food availability to optimize their growth (Zhu et al. 2015). Popova et al. (2021) observed that adult sand lizard (*Lacerta agilis*) preferred higher and denser herbaceous, while juveniles tend to select lower and denser grasses to minimize interspecific competition. Furthermore, given the limited defenses and small size of juveniles, they may be subject to intraspecific interactions, such as individual size, conspecific feeding, thereby dynamically adjusting habitat selection (Keren‐Rotem, Bouskila, and Geffen 2006; Claessen, de Roos, and Persson 2000). Delaney and Warner (2017) found that the habitat selection of juvenile brown anole lizard ( *Anolis sagrei* ) is influenced by adult density, body size, and other factors, including perch height, perch width, and substrate condition. In summary, individuals at different development stages have different physiological needs and are likely to exhibit differential habitat selection under the influence of external environmental pressures (Popova et al. 2021; Keren‐Rotem, Bouskila, and Geffen 2006).

Extensive research has been conducted on habitat selection in animals, including lizards. However, most previous studies on lizard have focused on single aspects, such as sex or age‐related differences, some crucial ecological factors in microhabitats, intraspecific or interspecific competition, the spatial and temporal factors, but few studies have considered a comprehensive range of factors (AlRashidi, Abdelgadir, and Shobrak 2021; Lortie et al. 2020; Zhu et al. 2015; Langkilde and Shine 2004; Ortega and Pérez‐Mellado 2016; Owen et al. 2024; Valdez Ovallez et al. 2023). The mountain dragon (*Diploderma splendidum*) serves as a notable case study in habitat selection due to its dense populations, pronounced sexual and age‐related variation, and consistent activity patterns. In this study, we quantified the specific habitat preferences of *D. splendidum* across different sex and age groups from multiple perspectives to elucidate their unique ecological requirements and analyze the key factors influencing the distribution in diverse habitats. This research not only enhances our understanding of the factors shaping organismal distribution across landscapes, but also provides theoretical reference for informing future reptile conservation and habitat management plans in the Jinsha River Basin.

## 2 Materials and Methods

### 2.1 Survey Region

This study was conducted in the lower Jinsha River valley, situated in Leibo County (28. 16° N, 103. 34° E), Sichuan Province, Western China. The region features a typical subtropical mountainous climate, characterized by distinct seasonal variations and notable diurnal temperature fluctuations. Annual average precipitation measures 900 mm, with approximately 1250–2600 h of sunshine each year. The lizards predominantly inhabit exposed rocks and sparse brush, with a high population density of approximately 10 lizards/ha. Vegetation primarily consists of shrubs (e.g., *Rumex acetosa* L. and *Trema cannabina dielsiana*) and small trees (e.g., *Leucaena leucocephala* , *Jacaranda mimosifolia* , *Ficus virens*, and *Dodonaea viscosa* ), interspersed with many planted forests (e.g., *Citrus reticulata* and *Zanthoxylum piasezkii*).

### 2.2 Individual Capture and Measurement

In 2021, a total of 125 *D. splendidum* lizards were captured using manual and lasso techniques during their peak activity periods from July to August between 10 a.m. and 04 p.m. Sex determination relied primarily on assessment of dorsal color patterns and the presence or absence of a hemipenis bulge. Snout‐vent length (SVL) was measured using a digital caliper (Ningbo Deli Tools Co. Ltd.) to nearest 0.01 mm. Based on reference to pertinent literatures and preliminary anatomical data from *D. splendidum* specimens, individuals with an SVL > 67 mm were classified as adult females, those with an SVL > 71 mm were classified as adult males, and the remaining individuals were categorized as juveniles (Wang et al. 2019, 2017). In accordance with the capture order, each lizard was assigned a unique identification number for tracking purposes, which was applied to their abdomen using nontoxic self‐inking acrylic paint that posed no physical harm (Wu et al. 2019). Capture location was marked with uniquely coded plastic cards to facilitate subsequent habitat data collection and release at the original capture sites. After collecting morphologic and habitat data, all lizards were released back into their respective capture sites.

### 2.3 Habitat Data Collection

Upon locating a lizard, a 5 × 5 m experimental plot was established, centered on the stationary point of lizard activity. Additionally, a random plot of the same size, representing potential available habitat, was randomly selected within surrounding area using the random riprap method. This method involves a stationary observer rotating 720° with closed eyes and dropping a stone to establish a 5 × 5 m plot centered on its landing location. To ensure precise measurements, light intensity indicators were promptly recorded after capturing each individual dragon lizard. After excluding data with significant errors and strictly adhering to matching rules, a total of 219 plots were selected, consisting of 94 random plots and 125 experimental plots. Eleven ecological factors were assessed in each plot using corresponding survey instruments, including seven numerical ecological factors and four categorical ecological factors (Table 1). The definition and classification of each ecological factor followed criteria outlined in previous studies (Yang et al. 2019; Farha et al. 2020; Table 1).

**TABLE 1:** Categorization of ecological factors associated with *Diploderma splendidum* was investigated within 5 × 5 m plots in Leibo in this study.

Ecological factor type	Ecological factor	Definition
Numerical factors	Tree height (cm)	Average height of trees within plots
	Perch height (cm)	Vertical elevation of arboreal habitat utilized by lizard, measured from ground level within plots
	Rock height (cm)	Average height of all rocks within plots
	Rock size (cm)	Average size of all rocks within plots
	Distance from nearest water (m)	Minimum horizontal distance from plot to water source (including springs, rivers, and other water bodies, without snow)
	Distance from nearest road (m)	Horizontal distance from plot to nearest road (including transporting, farming, or grazing)
	Light intensity (Lux)	Light intensity at capture point or centroid within plots
Categorical factors	Vegetation type	Main vegetation types were determined according to the vegetation composition of each plot and were divided into four types: Herb, Shrub and herb, Forest and herb, and Forest shrub and herb
	Vegetation density	Quantity of trees in plot with three categories: lower (< 1 m), middle (2–7 m), and upper (> 8 m)
	Vegetation coverage	Proportion of upper canopy coverage relative to ground surface in plot with three categories: lower (< 20%), middle (20%–60%), and upper (> 60%)
	Substrate status	Looseness of substrate in plot with three categories: loose (soil), general (soil‐macadam mix), firm (macadam) (contains more than 50% of substance)

### 2.4 Statistical Analyses

Before conducting statistical analyses, the Kolmogorov–Smirnov and Levene's tests were employed to assess data normality and homogeneity of variance, respectively.

To analyze the habitat selection preference of *D. splendidum* across different sex and age groups, Pearson's correlation analysis was conducted firstly to assess autocorrelation among numerical ecological factors. Kendall correlation analysis was conducted to assess autocorrelation among categorical ecological factors. Spearman correlation analysis was conducted to assess autocorrelation between numerical and categorical ecological factors. One of the factors exhibiting high autocorrelation (|*r*| > 0.8; Gardiner et al. 2015; Chiaverini et al. 2023) were subsequently excluded. Perch height, reflecting species‐specific preferences, was omitted from the importance of ecological factors in habitat selection preference analysis. After filtering the highly correlated data, a random forest model was constructed using these numerical and categorical factors, with ecological factors as the predictor variable and sample selection as the response variable. The random forest model initially employed 10‐fold cross‐validation of caret's train function to determine optimal values for mtry and ntree across different groups in R Studio (v23.3.1, Rаcinе 2012, Table S3). The optimal combinations for each group were as follows: adult male—mtry = 2, ntree = 500; adult female—mtry = 2, ntree = 800; juvenile male—mtry = 3, ntree = 500; and juvenile female—mtry = 2, ntree = 800. At this stage, the model demonstrated high accuracy and Kappa value indicating superior predictive performance. The minimum node size uses the default value of 1. Finally, based on determined values of mtry and ntree for each group in constructing the random forest model, we proceeded with analysis of ecological factor importance using Mean Decrease Gini index followed by generation of partial dependence diagrams (Zhang et al. 2020; R Development Core Team 2018).

To compare differences in habitat preference factors among different sex and age groups, a general linear model (GLM) was initially employed to quantify the effects of sex, age, and their interactions on habitat selection (Delaney and Warner 2016). Subsequently, simple effect analysis was conducted to evaluate the influence of ecological factor interactions, excluding nonsignificant interactions. For numerical factors conforming to a normal distribution, differences in habitat selection between different sex and age groups were assessed using independent sample *t*‐tests. In cases where numerical factors did not follow a normal distribution, differences in habitat selection among groups were assessed using the Mann–Whitney *U* test (Li et al. 2022). Chi‐square tests were performed to analyze disparities in categorical factors, such as slope direction and vegetation type, regarding sex and age preferences (Shilereyo et al. 2021). When significant differences were observed (*p* < 0.05), Vanderploeg and Scavia's selection index was employed to elucidate population‐specific habitat preferences.

All statistical analyses were conducted using Origin (v8.6), Excel (v2410), R Studio (v23.3.1, Rаcinе 2012), and SPSS software (v22.0).

## 3 Results

### 3.1 Habitat Preferences Across Different Sex and Age Groups

**FIGURE 1:**
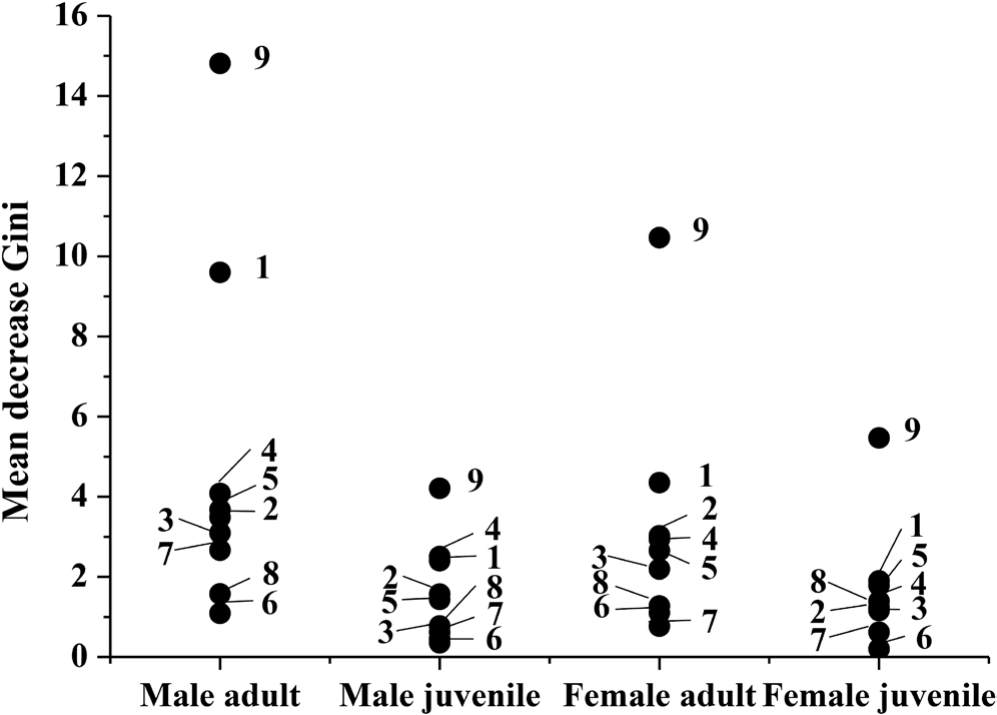
The relative importance of ecological factors in *Diploderma splendidum* varied across different sex and age groups for habitat selection. 1: Tree height (TH); 2: Distance from nearest water (DWS); 3: Distance from nearest road (DRS); 4: Light intensity (LI); 5: Rock height (RH); 6: Vegetation coverage (VC); 7: Vegetation density (VD); 8: Vegetation type (VT); 9: Substrate status (SS).

Autocorrelation analysis revealed a significant positive correlation between rock size and rock height (*r* = 0.970, Figure S1). Therefore, rock size was excluded from subsequent analyses to minimize estimates of uncertainty. The importance of variable in the random forest model for predicting *D. splendidum* habitat use, as determined by the mean decrease Gini index, are shown in Figure 1. Results identified substrate status (SS), tree height (TH), light intensity (LI), rock height (RH), and, to a lesser extent, vegetation coverage (VC), vegetation density (VD), and vegetation type (VT) as the primary factors influencing habitat selection among individuals of different sexes and ages (Figure 1).

**FIGURE 2:**
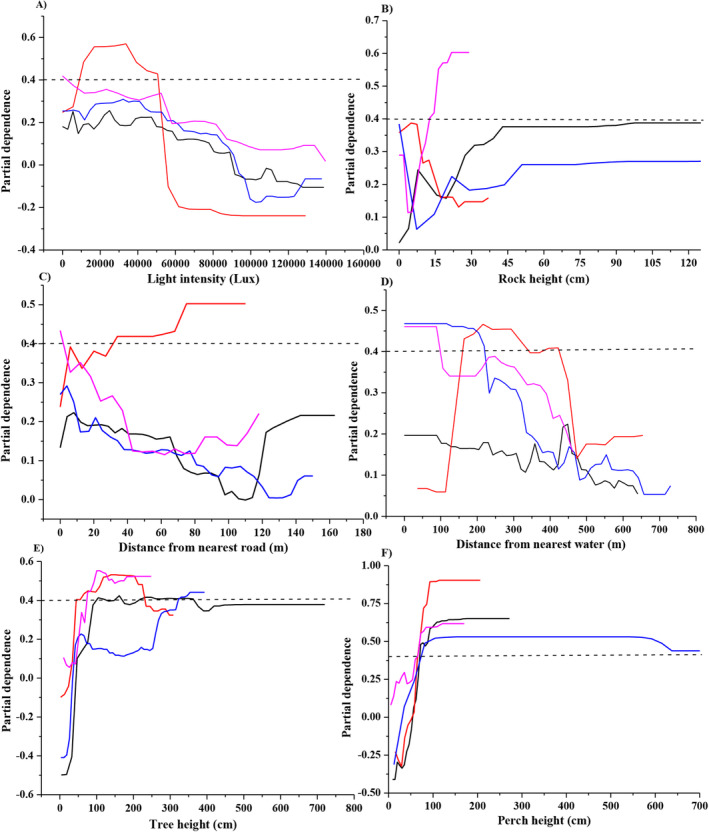
The partial dependence plots for each predictor variable in *Diploderma splendidum* across different sex and age groups. Y‐axes are partial dependence (dependence of probability of occurrence on one predictor variable after averaging out effects of other predictor variables in the model). Black: Adult males; Red: Juvenile males; Blue: Adult females; Purple: Juvenile females.

The partial dependence graph derived from the random forest model indicated a significantly increased probability of adult male occurrence when the tree height ranging from 90 to 350 cm, with a permanent stay height exceeding 67 cm (Figure 2E,F). Juvenile males were most likely to occur in habitats with tree heights ranging from 47 to 230 cm, minimum permanent stay height exceeding 65 cm, distances greater than 32 m from the nearest road, distances spanning from 160 to 427 m from the closest water sources, and light intensity ranging from 12,000 to 45,000 Lux (Figure 2A,C–F). Adult females were predominantly observed in habitats with tree heights over 340 cm, a minimum vegetation height of 62 cm, and distances from the nearest water sources < 225 m (Figure 2D–F). Juvenile females were more likely to be found in habitats with tree heights over 75 cm, the permanent stay height is > 60 cm, light intensity < 5000 lx, distance from nearest road source < 3 m, distances from nearest water source < 110 m, and rock heights > 12 cm (Figure 2).

### 3.2 Sex and Age‐Related Variations in Habitat Selection

**FIGURE 3:**
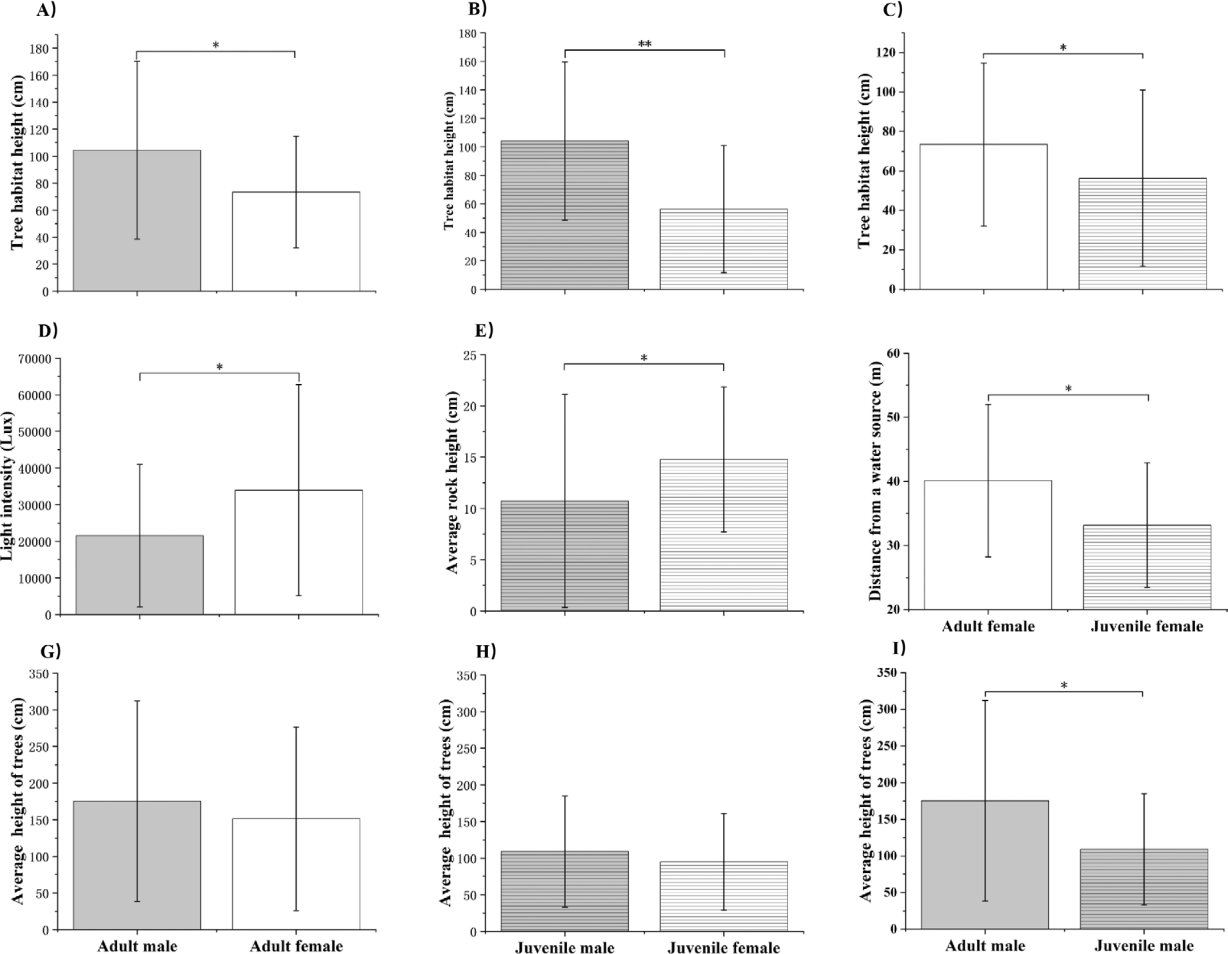
Selection and utilization of numerical variables in *Diploderma splendidum* across different sex and age groups. Light gray: Adult male; Empty white: Adult female; Light gray and line: Juvenile male; Empty white and line: Juvenile female. The vertical line is standard deviation (SD). The plot showed that “mean ±SD.” “**” indicates a highly significant difference, “*” indicates a significant difference.

Given the absence of interactions between different sexes and ages, subsequent analysis focused on univariate effects (Table S1). Compared to juveniles, adults showed a preference for higher trees (*F* = 0.183, *p* = 0.004) and sites more distant from water (*Z* = −2.276, *p* = 0.023). Compared to females, males showed a preference for higher tree positions (*Z* = −3.611, *p* = 0.000). Compared to adult females, adult males exhibited a preference for higher tree positions (*Z* = −2.216, *p* = 0.027, Figure 3A) and areas with lower light intensity (*Z* = −2.034, *p* = 0.042, Figure 3D) and moderate vegetation density (χ2 = 14.230, *p* = 0.001, Table 2). In comparison with juvenile females, juvenile males showed a preference for higher tree positions (*Z* = −2.75, *p* = 0.006, Figure 3B), low rock formations (*F* = −2.17, *p* = 0.03, Figure 3E; Table S2), and shrub and forest habitats over herbaceous habitats (χ2 = 8.842, *p* = 0.031, Table 2; Table S2).

**TABLE 2:** Selection and utilization of categorical ecological factors by individual preference in *Diploderma splendidum*.

Habitat type		Adult male (*n* = 52)		Adult female (*n* = 35)		Juvenile male (*n* = 18)		Juvenile female (*n* = 20)	
		Wi	Ei	Wi	Ei	Wi	Ei	Wi	Ei
Vegetation type	Herb	0.571	0.391	0.583	0.400	0.000	−1.000	0.625	0.429
	Shrub and herb	0.538	0.366	0.556	0.379	0.600	0.412	0.769	0.509
	Forest and herb	0.559	0.382	0.56	0.383	0.600	0.412	0.4	0.231
	Forest shrub and herb	0.579	0.397	0.571	0.391	0.600	0.412	0.5	0.333
Vegetation coverage	Lower	0.500	0.200	1.000	0.500	1.000	0.500	0.000	−1.000
	Middle	0.559	0.254	0.475	0.176	0.476	0.177	0.625	0.305
	Upper	0.567	0.26	0.684	0.345	0.778	0.400	0.556	0.250
Vegetation density	Lower	0.029	0.029	0.568	0.260	0.615	0.298	0.529	0.228
	Middle	0.385	0.385	0.545	0.242	0.500	0.200	0.750	0.385
	Upper	0.259	0.259	0.571	0.264	0.625	0.305	0.625	0.305
Substrate status	Loose	0.429	0.125	0.500	0.200	0.000	−1.000	1.000	0.500
	General	0.565	0.258	0.576	0.268	0.552	0.247	0.586	0.275
	Firm	1.000	0.500	0.000	−1.000	1.000	0.500	0.667	0.334

*Notes:* Ei, selection index; Wi, selectivity coefficient.

Compared to juvenile females, adult females exhibited a preference for higher tree positions (*Z* = −2.031, *p* = 0.042, Figure 3C) and sites more distant from water (*F* = −2.051, *p* = 0.040, Figure 3F). In comparison with juvenile males, adult males displayed a preference for elevated tree habitats (*F* = −2.002, *p* = 0.045, Figure 3I). Additionally, adults favored habitats with general or firm substrate status (χ2 = 15.082, *p* = 0.001, Table 2; Table S2).

## 4 Discussion

The habitat selection of wildlife is the result of long‐term evolutionary adaptation, influenced by a variety of intrinsic and external factors (Zhu et al. 2015; Reunanen, Mönkkönen, and Nikula 2002). In this study, we investigated the patterns of habitat selection in a mountain dragon from Western China across different sex and age groups. Our findings revealed significant differences between adults and juveniles regarding substrate condition, vegetation height, perch height, and distance from water. Furthermore, females and males exhibited dissimilarities in their utilization of vegetation density, perch height, light intensity, and rock height. Our study demonstrates complex variations in habitat selection by *D. splendidum* that are dependent on age and sex. Similar variations were observed among different sexes and ages in microhabitat and macrohabitat selection within in the arboreal lizard *Anolis sagrei* (Delaney and Warner 2016).

The observed differences in habitat selection among individuals of different sexes may be attributed to variations in physiological structure, living habits, and behavior (Conde et al. 2010; Eifler, Eifler, and Eifler 2007). Adult males generally exhibited a preference for elevated habitats within moderately vegetated environments, similar to the preference for higher tree perches observed in territorial lizards such as *Anolis sagrei* (Delaney and Warner 2016). Male lizards tend to occupy higher tree positions to enhance visibility for guarding territory, engaging in interspecific communication, displaying behavior, and foraging (Song et al. 2017). However, given the greater threats inherent in occupying higher positions, males also prefer habitats with denser vegetation to reduce their risk exposure (Browne and Paszkowski 2014; Robert, Fletcher, and Miller 2006). Females, especially pregnant females, tend to prefer areas with higher safety indices due to their significant investment in reproduction (Eifler, Eifler, and Brown 2012; Jerosch et al. 2018). Our results indicated that females preferred lower tree positions or higher rocks, which provide better opportunities to escape from predators, and reduce risks associated with slower mobility, similar to the preference for lower perches and higher foraging observed in female arboreal lizards such as *Anolis polylepis* (Andrews 1971). Lizards are ectotherms and their physiology is influenced by external conditions, especially light and temperature (Tan et al. 2019). Notably, we found that adult females preferred higher light intensity, likely to elevate their body temperature, enhance physiological and biochemical responses, and promote gonadal development, thereby influencing habitat selection decisions (Harvey and Weatherhead 2010; Melville and SchulteII 2001). Consistent with previous studies on female blunt‐nosed leopard lizard ( *Gambelia sila* ), which have shown increased surface activity in denser vegetation, adult female *D. splendidum* also displayed a preference for higher vegetation densities. This preference is likely attributed to enhance resources availability and shelter options, potentially facilitating thermoregulation (Melville and SchulteII 2001; Castilla and Bauwens 1992; Westphal et al. 2018).

The differences in habitat selection among individuals of varying ages may also be related to their developmental and physiological diversity (Ficetola, Pennati, and Manenti 2013). Our findings indicated that adult lizards typically preferred habitats with taller vegetation, which offer more abundant food sources, better concealment, and an extensive canopy that assists in thermoregulation (Castilla and Bauwens 1992). Vegetation characteristics in microhabitats have been shown to facilitate thermoregulation in animals (Bradley et al. 2022; Decencière et al. 2024; Schultz 2024; Zhu et al. 2015). Similarly, previous studies have revealed that preference differences between adults and juveniles may help reduce ecological niche competition and cannibalism, especially under high population densities (Keren‐Rotem, Bouskila, and Geffen 2006). Our results indicated that juvenile males, facing higher predation risks, tended to select larger rocky shelters, shrubs, and ground‐level sites in forested areas. This behavior helps reduce daytime visual predator tracking, increase the availability of retreat sites, and lower predation risk (Niewiarowski et al. 1997). Similarly, Keren‐Rotem, Bouskila, and Geffen (2006) found that the juvenile common chameleons ( *Chamaeleo chamaeleon* ) prefer low grasslands for concealment, while adults favor shrubs and trees. Our results also demonstrated that adults preferred general or compacted substrate conditions, similar to the habitat selection observed in rock lizards (*Iberolacerta bonnali*) (Arribas 2009). This preference aligns with the idea that both species select habitats that minimize negative impacts, thereby facilitating escape or enhancing predation rates (Song et al. 2017; Nemes et al. 2006; Mackey 2010).

Our results revealed significant differences in habitat selection among lizards based on sex and age classes, contributing to existing knowledge on reptile habitat selection and enhancing our understanding of the distribution and specific characteristics of these lizards. Furthermore, our results can serve as a scientific foundation and research paradigm for lizard conservation or other species inhabiting the arid and hot river valleys of the Hengduan Mountain region, such as the development of targeted conservation plans, delineation of conservation areas, and the promotion of habitat restoration measures with regard to the species' habitat preferences and the importance of ecological factors. However, the low occurrence rate of lizards during winter and early spring in the study area limited data collection. Consequently, only habitat selection data from the breeding period were analyzed, as year‐round data collection was not feasible. It is important to note that habitat selection may vary across seasons, and factors such as temporal migration and interspecific interactions could influence specific habitat preferences of individuals. Therefore, future research will continue to monitor habitat changes influenced by various factors at different scales to address and resolve these uncertainties.

## 5 Conclusions

In conclusion, this study identified tree height, perch height, light intensity, vegetation density, vegetation type, and substrate status as the primary determinants of habitat selection for the mountain dragon lizard in the hot‐dry river valley ecosystem of the lower Jinsha River Basin. Other environmental factors and interspecific interference were not found to be limiting resources. The findings also indicated that the different age and sex groups of *D. splendidum* have different habitat preferences. For example, adults preferred general or compacted substrate conditions and habitats with taller vegetation; male lizards tend to occupy higher tree positions and habitats with denser vegetation, while females preferred lower tree positions or higher rocks. These findings suggest that the mountain dragon lizard may serve as a valuable environmental indicator for ecological changes and conservation efforts. The results of this study can provide some information for the development of animal conservation programs, and also provide an example for ecological studies in the dry and hot valley region of the Jinsha River Basin. Furthermore, further research is needed to determine the sensitivity of this species to the temporal migration.

## Author Contributions

**Dongqing Zheng:** data curation (equal), investigation (equal), methodology (equal), software (equal), writing – original draft (lead). **Ling Li:** methodology (equal), software (equal), writing – original draft (equal). **Wei Gao:** funding acquisition (equal), visualization (equal), writing – review and editing (equal). **Meiqi Chen:** data curation (equal), investigation (equal), software (equal). **Peng Guo:** funding acquisition (equal), visualization (equal), writing – review and editing (equal). **Yayong Wu:** funding acquisition (equal), visualization (equal), writing – review and editing (equal).

## Conflicts of Interest

The authors declare no conflicts of interest.

## Supporting information


**Figure S1.** Autocorrelation analysis of habitat factors in *Diploderma splendidum*. DRS, distance from nearest road; DWS, distance from nearest water; LI, light intensity; RH, rock height; RS, Rock size; SS, substrate status; TH, tree height; VC, vegetation coverage; VD, vegetation density; VT, vegetation type.

**Table S1.** The interactions of ecological factors in different age and sex groups of *Diploderma splendidum*.

**Table S2.** A chi‐square test of classified ecological factors in different age and sex groups of *Diploderma splendidum*.

**Table S3.** A 10‐fold cross‐validated resampling of the random forest model in different age and sex groups of *Diploderma splendidum*.

Appendix S1.

Appendix S2.

## Data Availability

All raw data and analysis code are stored in DRYAD (DOI:10.5061/dryad. mcvdnck87). In addition, we provide a “Private for Peer Review” link: https://datadryad.org/stash/share/GQGRiqJIpdxC5eoEBhize4LSPtF9fjS40XQe2mYd‐‐0. All analyses were performed with publicly available programs. All analysis codes can be publicly accessed and utilized.
