# Supplementary material for: Sex and Age Differences in Habitat Selection of the Mountain Dragon Lizard (Diploderma splendidum) From Western China
Source: Ecol Evol. 2024 Dec 23;14(12):e70724. doi: 10.1002/ece3.70724 (PMC11664211; doi:10.1002/ece3.70724)
Supplement: Supplementary file 1 — Figure S1. Autocorrelation analysis of habitat factors in Diploderma splendidum. DRS, distance from nearest road; DWS, distance from nearest water; LI, light intensity; RH, rock height; RS, Rock size; SS, substrate status; TH, tree height; VC, vegetation coverage; VD, vegetation density; VT, vegetation type. [file ECE3-14-e70724-s005.docx]

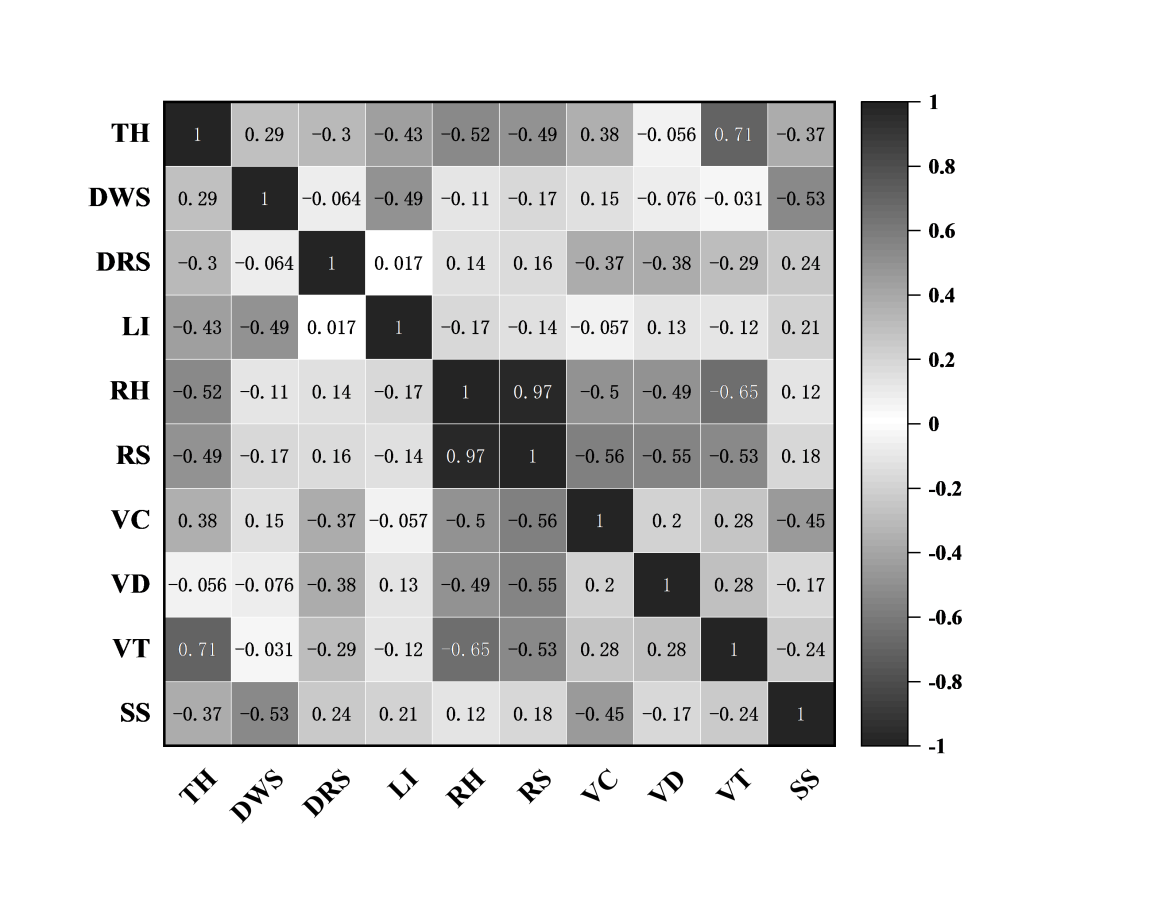


Figure S1 Autocorrelation analysis of habitat factors in *Diploderma splendidum*. TH: tree height; DWS: distance from nearest water; DRS: distance from nearest road; LI: light intensity; RH: rock height; RS: Rock size; VT: vegetation type; VD: vegetation density; VC: vegetation coverage; SS: substrate status.
