# Supplementary material for: Sex and Age Differences in Habitat Selection of the Mountain Dragon Lizard (Diploderma splendidum) From Western China
Source: Ecol Evol. 2024 Dec 23;14(12):e70724. doi: 10.1002/ece3.70724 (PMC11664211; doi:10.1002/ece3.70724)
Supplement: Supplementary file 2 — Table S1. The interactions of ecological factors in different age and sex groups of Diploderma splendidum. [file ECE3-14-e70724-s006.docx]

Table S1 The interactions of ecological factors in different age and sex groups of *Diploderma splendidum*

| **Variables** | ***df*** | ***F* value** | ***P* value** |
| --- | --- | --- | --- |
| **Numerical factors** |  |  |  |
| Tree height (cm) |  |  |  |
| Age class | 1 | 7.346 | **0.007** |
| Sex | 1 | 0.716 | 0.398 |
| Age class*Sex | 1 | 0.046 | 0.831 |
| Perch height (cm) |  |  |  |
| Age class | 1 | 0.668 | 0.414 |
| Sex | 1 | 13.703 | **0.000** |
| Age class*Sex | 1 | 0.621 | 0.431 |
| Rock height (cm) |  |  |  |
| Age class | 1 | 1.675 | 0.196 |
| Sex | 1 | 1.461 | 0.227 |
| Age class*Sex | 1 | 0.401 | 0.526 |
| Rock size (cm) |  |  |  |
| Age class | 1 | 0.844 | 0.358 |
| Sex | 1 | 0.338 | 0.561 |
| Age class*Sex | 1 | 1.224 | 0.269 |
| Distance from nearest water source (m) |  |  |  |
| Age class | 1 | 6.604 | **0.010** |
| Sex | 1 | 1.185 | 0.276 |
| Age class*Sex | 1 | 0.541 | 0.462 |
| Distance from nearest road source (m) |  |  |  |
| Age class | 1 | 0.435 | 0.509 |
| Sex | 1 | 0.330 | 0.565 |
| Age class*Sex | 1 | 0.000 | 0.994 |
| Light intensity (Lux) |  |  |  |
| Age class | 1 | 0.019 | 0.891 |
| Sex | 1 | 4.833 | **0.028** |
| Age class*Sex | 1 | 0.111 | 0.738 |
| **Classified factors** |  |  |  |
| Vegetation type |  |  |  |
| Age class | 1 | 0.002 | 0.966 |
| Sex | 1 | 0.161 | 0.688 |
| Age class*Sex | 1 | 0.141 | 0.707 |
| Vegetation density |  |  |  |
| Age class | 1 | 0.019 | 0.892 |
| Sex | 1 | 2.529 | 0.112 |
| Age class*Sex | 1 | 1.001 | 0.317 |
| Vegetation coverage |  |  |  |
| Age class | 1 | 0.258 | 0.611 |
| Sex | 1 | 7.816 | **0.005** |
| Age class*Sex | 1 | 2.661 | 0.103 |
| Substrate status |  |  |  |
| Age class | 1 | 4.409 | **0.036** |
| Sex | 1 | 0.222 | 0.637 |
| Age class*Sex | 1 | 0.427 | 0.513 |

**Note**: The bold number represents the *P* value less than 0.05.
