# Supplementary material for: Sex and Age Differences in Habitat Selection of the Mountain Dragon Lizard (Diploderma splendidum) From Western China
Source: Ecol Evol. 2024 Dec 23;14(12):e70724. doi: 10.1002/ece3.70724 (PMC11664211; doi:10.1002/ece3.70724)
Supplement: Supplementary file 3 — Table S2. A chi‐square test of classified ecological factors in different age and sex groups of Diploderma splendidum. [file ECE3-14-e70724-s002.docx]

Table S2 A chi-square test of classified ecological factors in different age and sex groups of *Diploderma splendidum*

|  | Variables | *df* | χ2 value | | *P* value |
| --- | --- | --- | --- | --- | --- |
| Adult male/Adult female | Vegetation type | 3 | | 1.493 | 0.684 |
|  | Vegetation coverage | 2 | | 1.228 | 0.541 |
|  | Vegetation density | 2 | | 14.23 | **0.001** |
|  | Substrate status | 2 | | 1.111 | 0.574 |
| Juvenile male/Juvenile female | Vegetation type | 3 | | 8.842 | **0.031** |
|  | Vegetation coverage | 2 | | 2.234 | 0.327 |
|  | Vegetation density | 2 | | 0.145 | 0.930 |
|  | Substrate status | 2 | | 0.928 | 0.629 |
| Adult male/Juvenile male | Vegetation type | 3 | | 3.702 | 0.296 |
|  | Vegetation coverage | 2 | | 0.377 | 0.828 |
|  | Vegetation density | 2 | | 2.252 | 0.324 |
|  | Substrate status | 2 | | 3.689 | 0.158 |
| Adult female/Juvenile female | Vegetation type | 3 | | 3.887 | 0.274 |
|  | Vegetation coverage | 2 | | 3.171 | 0.205 |
|  | Vegetation density | 2 | | 1.514 | 0.469 |
|  | Substrate status | 2 | | 3.863 | 0.145 |
| Adult/Juvenile | Vegetation type | 3 | | 2.902 | 0.407 |
|  | Vegetation coverage | 2 | | 0.757 | 0.685 |
|  | Vegetation density | 2 | | 0.465 | 0.793 |
|  | Substrate status | 2 | | 15.082 | **0.001** |
| Male/Female | Vegetation type | 3 | | 5.517 | 0.138 |
|  | Vegetation coverage | 2 | | 0.146 | 0.930 |
|  | Vegetation density | 2 | | 11.441 | **0.003** |
|  | Substrate status | 2 | | 0.071 | 0.965 |

**Note**: The bold number represents the *P* value less than 0.05.
