# Supplementary material for: Sex and Age Differences in Habitat Selection of the Mountain Dragon Lizard (Diploderma splendidum) From Western China
Source: Ecol Evol. 2024 Dec 23;14(12):e70724. doi: 10.1002/ece3.70724 (PMC11664211; doi:10.1002/ece3.70724)
Supplement: Supplementary file 4 — Table S3. A 10‐fold cross‐validated resampling of the random forest model in different age and sex groups of Diploderma splendidum. [file ECE3-14-e70724-s004.docx]

Table S3 A 10-fold cross-validated resampling of the random forest model in different age and sex groups of *Diploderma splendidum*

|  |  |  |  | 10-fold cross-validated resampling | | | | | | |  |  |  |  |
| --- | --- | --- | --- | --- | --- | --- | --- | --- | --- | --- | --- | --- | --- | --- |
|  | Adult male |  |  |  | Juvenile male |  |  |  | Adult female |  |  |  | Juvenile female |  |
| mtry | Accuracy | Kappa |  | mtry | Accuracy | Kappa |  | mtry | Accuracy | Kappa |  | mtry | Accuracy | Kappa |
| **2** | **0.924444** | **0.844002** |  | 2 | 0.825 | 0.65 |  | **2** | **0.904762** | **0.796273** |  | **2** | **0.966667** | **0.94** |
| 3 | 0.892222 | 0.781145 |  | **3** | **0.866667** | **0.74** |  | 3 | 0.890476 | 0.768375 |  | 3 | 0.933333 | 0.84 |
| 4 | 0.892222 | 0.781145 |  | 4 | 0.833333 | 0.7 |  | 4 | 0.890476 | 0.768375 |  | 4 | 0.9 | 0.78 |
| 5 | 0.881111 | 0.759222 |  | 5 | 0.833333 | 0.7 |  | 5 | 0.890476 | 0.768375 |  | 5 | 0.9 | 0.78 |
| 6 | 0.881111 | 0.759222 |  | 6 | 0.833333 | 0.7 |  | 6 | 0.890476 | 0.768375 |  | 6 | 0.866667 | 0.74 |
| 7 | 0.892222 | 0.782299 |  | 7 | 0.833333 | 0.7 |  | 7 | 0.890476 | 0.768375 |  | 7 | 0.866667 | 0.74 |
| 8 | 0.892222 | 0.782299 |  | 8 | 0.833333 | 0.7 |  | 8 | 0.904762 | 0.79881 |  | 8 | 0.866667 | 0.74 |
| 9 | 0.892222 | 0.782299 |  | 9 | 0.833333 | 0.7 |  | 9 | 0.904762 | 0.79881 |  | 9 | 0.866667 | 0.74 |
|  | ntree = 500 |  |  |  | ntree = 500 |  |  |  | ntree = 800 |  |  |  | ntree = 800 |  |
| mtry | Accuracy | Kappa |  | mtry | Accuracy | Kappa |  | mtry | Accuracy | Kappa |  | mtry | Accuracy | Kappa |
| 2 | 0.915556 | 0.827511 |  | 2 | 0.833333 | 0.7 |  | 2 | 0.870476 | 0.728205 |  | 2 | 0.966667 | 0.94 |
| 9 | 0.903333 | 0.806393 |  | 8 | 0.858333 | 0.75 |  | 9 | 0.876191 | 0.738667 |  | 8 | 0.866667 | 0.74 |
| 16 | 0.903333 | 0.806393 |  | 15 | 0.825 | 0.69 |  | 16 | 0.890476 | 0.77503 |  | 15 | 0.841667 | 0.69 |
